# Education is associated with lower levels of abdominal obesity in women with a non-agricultural occupation: an interaction study using China’s four provinces survey

**DOI:** 10.1186/1471-2458-13-769

**Published:** 2013-08-21

**Authors:** Amina Aitsi-Selmi, Ruoling Chen, Martin J Shipley, Michael G Marmot

**Affiliations:** 1 Department of Epidemiology and Public Health, 1-19, Torrington Place, London WC1E 6BT, UK; 2 Division of Health and Social Care Research, King’s College London, 7th Floor, Capital House 42, Weston Street, London SE1 3QD, UK

**Keywords:** Socioeconomic status, Obesity, Low- and middle-income countries, Epidemiology, Women, China, Education, Occupation, Waist circumference, Transition

## Abstract

**Background:**

The prevalence of obesity is increasing rapidly in low- and middle-income countries (LMICs) as their populations become exposed to obesogenic environments. The transition from an agrarian to an industrial and service-based economy results in important lifestyle changes. Yet different socioeconomic groups may experience and respond to these changes differently. Investigating the socioeconomic distribution of obesity in LMICs is key to understanding the causes of obesity but the field is limited by the scarcity of data and a uni-dimensional approach to socioeconomic status (SES). This study splits socioeconomic status into two dimensions to investigate how educated women may have lower levels of obesity in a context where labour market opportunities have shifted away from agriculture to other forms of employment.

**Methods:**

The Four Provinces Study in China 2008/09 is a household-based community survey of 4,314 people aged ≥60  years (2,465 women). It was used to investigate an interaction between education (none/any) and occupation (agricultural/non-agricultural) on high-risk central obesity defined as a waist circumference ≥80 cm. An interaction term between education and occupation was incorporated in a multivariate logistic regression model, and the estimates adjusted for age, parity, urban/rural residence and health behaviours (smoking, alcohol, meat and fruit & vegetable consumption). Complete case analyses were undertaken and results confirmed using multiple imputation to impute missing data.

**Results:**

An interaction between occupation and education was present (*P* = 0.02). In the group with no education, the odds of central obesity in the sedentary occupation group were more than double those of the agricultural occupation group even after taking age group and parity into account (OR; 95%CI: 2.21; 1.52, 3.21), while in the group with any education there was no evidence of such a relationship (OR; 95%CI: 1.25; 0.92, 1.70). Health behaviours appeared to account for some of the association.

**Conclusion:**

These findings suggest that education may have a protective role in women against the higher odds of obesity associated with occupational shifts in middle-income countries, and that investment in women’s education may present an important long term investment in obesity prevention. Further research could elucidate the mechanisms behind this association.

## Background

Non-communicable diseases and their risk factors, including obesity, account for the largest proportion of mortality and morbidity in the world today [1,2] and are a growing burden in lower income countries [3]. Between 1980 and 2008, the global age-standardised prevalence of obesity rose from 7.9 to 13.8% in women and 4.8 to 9.8% in men [4]. Obesity is linked to a life-long risk of several major chronic diseases including cardiovascular diseases, type 2 diabetes, selected cancers, asthma, gallbladder disease, osteoarthritis and chronic back pain [5] as well as lower cognitive function in the elderly [6].

Occupational change is an important social determinant of obesity risk as lower income countries develop [7]. Transition from an agrarian to an industrial and/or service-based economy changes conditions of daily living [8,9]. This occupational shift is usually associated with migration from rural to urban environments where diets include a greater proportion of fat and sugar [10] and the physical environment is more conducive to obesity [11,12]. Therefore, the association between occupation and obesity tends to be positive in low- and middle-income countries.

The association with education is less consistent and may depend on the level of economic development of the country [13,14]. Education is well known to be beneficial to health and may protect against obesity through cognitive advantages that result in healthier lifestyles [15-17]. This implies that occupation and education may act differently in relation to obesity risk as countries develop economically. Yet investigations into these possibilities are few due to methodological and data limitations as well as the relative novelty of research into socioeconomic inequalities in non-communicable diseases in low- and middle-income countries.

China has undergone rapid economic growth since the start of reforms in 1978 with many people moving to work in the manufacturing and service sectors as a result of the move away from an agrarian to an urban-based economy [18,19]. In parallel, changes in physical activity levels and diet have taken place and a concurrent dramatic increase in central obesity prevalence has been observed from 8.5 to 27.8% in women between 1993 and 2009 [10,20-22]. Increased levels of obesity have been observed in both urban and rural Chinese populations with women displaying higher rates of obesity than men [22,23]. There are some reports that higher education might protect against obesity in Chinese women [22], at least in urban areas [21] but none have investigated this specifically nor examined the relationship with other markers of socio-economic status such as occupation.

In this study we examine the interaction between education and occupational status in relation to central obesity in a large scale community-based survey in China, to investigate the hypothesis that having a non-agricultural occupation will be associated with higher odds of obesity compared with having an agricultural occupation, and that education might protect against this. In other words, the study examines whether education modifies the association between occupational status and obesity in women.

## Methods

### Participants

The study population for this analysis was derived from participants in the Four Provinces study of dementia [24]. The four provinces (Guangdong, Heilongjiang, Shanghai and Shanxi) were selected to be nationally representative and compare to other provinces in China in terms of economic development. Randomised cluster sampling was employed to choose residential communities from within each of the four provinces (detailed location data available on request) between 2008 and 2009. One rural and one urban community from each of the four provinces were selected, with the aim of recruiting no fewer than 500 participants in each community. The target population consisted of those residents aged 60 years and over who had lived in the selected areas for at least five years. The participant characteristics for each province are shown in Table 1.

**Table 1:** Participant characteristic by province, Four Provinces study, China (2008/09)

	**Guangdong**	**Shanghai**	**Heilongjiang**	**Shanxi**
	**(N = 440)**	**(N = 630)**	**(N = 523)**	**(N = 328)**
	**n (%)**	**n (%)**	**n (%)**	**n (%)**
WC (cm)				
Not centrally obese^1^	199 (45.2)	191 (30.3)	146 (27.9)	116 (35.4)
Centrally obese^2^	241 (54.8)	439 (69.7)	377 (72.0)	212 (64.6)
Education level				
None	284 (64.5)	313 (49.7)	242 (46.3)	110 (33.5)
Any	156 (35.5)	317 (50.3)	281 (53.7)	218 (66.5)
Occupation group				
Agricultural	360 (81.8)	267 (42.4)	255 (48.8)	166 (50.6)
Non-agricultural	80 (18.9)	363 (57.6)	268 (51.2)	162 (49.4)
Age group				
60-69	182 (41.4)	289 (45.9)	216 (41.3)	188 (57.3)
70-79	171 (38.9)	246 (39.0)	209 (40.0)	123 (37.5)
80+	87 (19.8)	95 (15.1)	98 (18.7)	17 (5.2)
Area of residence				
Urban	198 (45.0)	358 (56.8)	279 (53.4)	161 (49.1)
Rural	242 (55.0)	272 (43.2)	244 (46.7)	167 (50.9)
Parity				
0	1 (0.23)	5 (0.8)	23 (4.4)	0
1-3	120 (27.3)	482 (76.5)	191 (36.5)	159 (48.5)
4+	319 (72.5)	143 (22.7)	309 (59.1)	169 (51.5)

**Figure 1:**
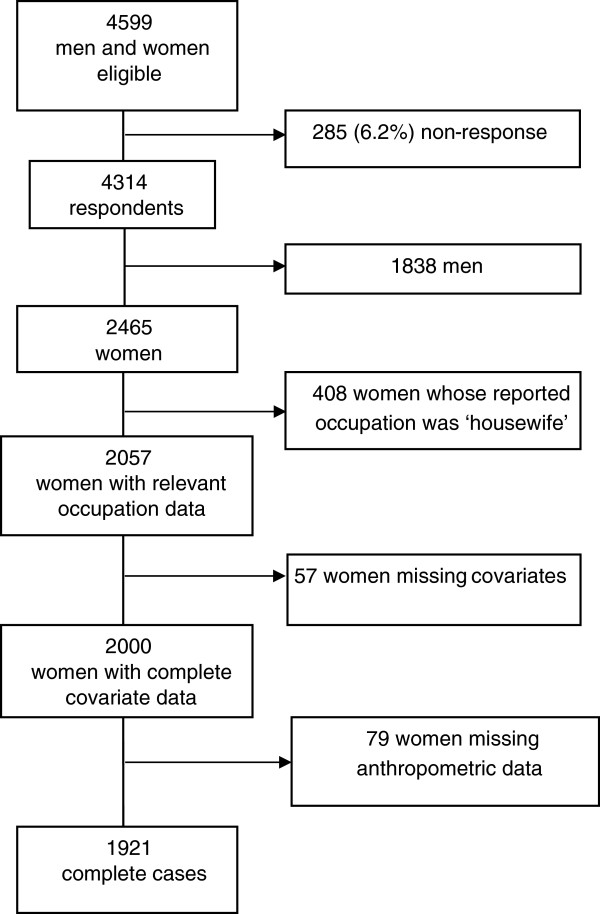
Participant selection for the analysis from the Four Provinces study.

A total of 4,314 participants were recruited of which 2,465 were women, representing an overall response rate of 93.8%. The participants were interviewed at home by trained survey teams using locally validated instruments including a general health and risk factor questionnaire [25]. Of the 2,465 women included in the survey, 408 women whose occupation was reported as ‘housewife’ were excluded for the purposes of the analysis leaving 2,057 women. Of the 2,057 women, 1,921 (93.4%) had complete covariate and anthropometric data, and 136 (6.6%) were missing those data. Figure 1 gives a full description of the participants included in the analytic sample.

### Outcome

Waist circumference (WC) was measured for all participants, according to standard procedures, with a plastic tape-measure placed mid-way between the lowest rib and the iliac crest and within 0.1 cm [26]. The cut-off point was defined according to the International Diabetes Federation (high risk central obesity: WC ≥80 cm in women) [27]. This accounts for the considerably higher percentage body fat present at sex and age equivalent BMIs [28], and the higher disease risk at lower levels of WC compared with Caucasians [29,30].

### Socio-economic status and other risk factors variables

The participants’ education level and main occupation were recorded using a health and risk factor questionnaire [24]. Education level was coded in two categories: 1 = no education, 2 = any education (including primary, secondary and higher education), as significant cognitive advantages are likely to exist when comparing those who have some level of education with those who are illiterate regardless of the work sector. The occupation variable was based on the participants’ reported longest employment and divided into two categories: 1 = agricultural (reported in the survey as ‘peasant’); 2 = non-agricultural. The latter category included manual workers (55% of the total) and office-based workers in administration, teaching and sales.

**Figure 2:**
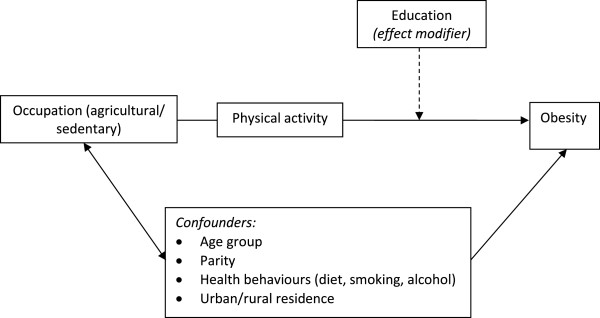
Simplified path diagram of the associations between education, occupation and obesity.

A number of covariates were included in the multivariate model (see Figure 2). Age was categorised into ten year age bands (60-69 yrs = 1; 70-79 yrs = 2; 80 + = 3). Health behaviours were self-reported and included current alcohol consumption (no/yes), smoking status (no/yes), meat consumption (less than once a day/once a day or more) and fruit & vegetable consumption (less than once a day/once a day or more). Area of residence was based on the administrative definition used in the sampling strategy (urban/rural) and used as a crude proxy for environmental factors.

### Statistical analysis

All analyses were conducted using the Stata 12SE® statistical package. Sampling characteristics and the prevalence of central obesity for each participant characteristic were calculated. The unadjusted and adjusted odds ratios for obesity and their 95% confidence intervals (OR; 95%CI) comparing the higher and lower socioeconomic groups were estimated for each of education (any *vs.* none) and occupation (non-agricultural *vs.* agricultural). A logistic regression model was used to estimate the separate and joint effects of the socioeconomic status (SES) variables on obesity. The separate effects were calculated based on a model that included either education or occupation as the main exposure. The joint effects of education and occupation on obesity were estimated by including an interaction term between education and occupation in the model.

First, from the model including the occupation by education interaction term, the effect of occupation (non-agricultural *vs.* agricultural) was estimated for the group with no education for the group with no education (main effect of occupation). Then, using the interaction term, using the interaction term estimated from the model and the main effect of occupation was calculated for the group with education, thus describing how education modifies the association between occupation and excess adiposity. In order to fully illustrate the interaction, the odds of central obesity. The odds of central adiposity were also calculated for each combination of education level and occupation relative to the odds in the referent category ‘education level = none and occupation = agricultural. This estimation was performed using logistic regression to produce the ORs adjusted for age group parity, area of residence and health behaviours and the results presented in a graph.

Data missingness in the Four Provinces study was investigated, by examining women who had complete covariate data and comparing those with and without anthropometric data (N = 1,921 *vs*. N = 79 respectively). Chi-squared tests showed statistically significant differences for more than half of the variables to be included in the regression model. Thus, a decision was made to impute the missing data in order to assess any potential bias in the complete case analyses. The Stata 12SE® chained equations command was used to perform multiple imputation and the imputations were performed using all the variables to be incorporated in the final model including education, occupation, age group, parity, area of residence, the health behaviour variables and the outcome variable. A marital status variable available in the dataset was investigated as an auxiliary variable in the imputation, but was discarded as there was no correlation with central obesity in a multivariate model with missing/non-missing WC data as the outcome. Twenty datasets were imputed and estimates from analyses on these datasets were averaged using Rubin’s rules [31]. All analyses were conducted comparing both the complete case analyses and the analyses using multiple imputation.

The interaction term in the regression model for the complete cases was examined for significance using Likelihood Ratio (LR) testing, comparing the model without an interaction term nested within the model with the interaction term. As this test was not valid with the imputed data, the *P*-value for the Wald test was used instead, with the null hypothesis being that the interaction term equals zero. Some authors have suggested that logistic regression is unsuitable for use when the outcome prevalence is common (>10%) [32], proposing a modified Poisson or log binomial model instead. However, in this particular study, logistic regression was justified for a number of reasons including the cross-sectional nature of the data and for ease of comparison with other studies [33].

### Ethical review

The use of the Four Provinces data was approved by Dr Ruoling Chen who holds the dataset at King’s College London. The study was considered exempt from full review by University College London because the study is based on an anonymous, public-use dataset with no identifiable information on the survey participants. The Chinese Four Provinces study is approved by the national body that approves research studies on humans in China, and written consent was obtained by the interviewers from each participant.

## Results

The analysis showed that participants who had complete covariate data but were missing anthropometric data (N = 79) were more likely to live in urban areas, have no education and report an agricultural occupation. Multiple imputation resulted in the inclusion of missing waist circumference data (n = 79) and the missing covariates (n = 57). These cases, added to the number of participants with complete anthropometric and covariate data (n = 1,921), resulted in a fully imputed sample of 2,057 participants. The results from the complete case analyses and the multiple imputation analyses were very similar and, therefore, only the results of the complete case dataset are shown in the following sections.

### Sociodemographic characteristics and central obesity prevalence

Table 1 shows that the four provinces were broadly comparable although there was some variability in obesity levels. Table 2 summarises the characteristics of the participants and the prevalence of obesity by characteristic. It shows that two thirds of the women were classified as centrally obese. About 49.4% reported having no education and 50.6% reported agricultural work as their longest held occupation. When examining occupation subgroups by education level, 80.3% (S.E.: 1.3) of women with no education reported having an agricultural occupation and 70.6% (S.E.: 1.5) with any education reported having a non-agricultural occupation. The prevalence of central obesity was greater in urban areas than in rural areas, in women with no children, those with any education, and those with a non-agricultural occupation. The prevalence of obesity by education and occupation subgroup showed that the difference in prevalence between those who worked in non-agricultural compared with agricultural occupations was greater, and in the opposite direction, in women with no education. In terms of health behaviours, statistically significant differences were present for smoking status and meat consumption. In terms of SES the group with the highest prevalence of central obesity was in the subgroup of women who had both no education and a non-agricultural occupation (77.5% S.E.: 3.1).

**Table 2:** Participant characteristics and prevalence of central obesity, Four Provinces Study, China (2008/09)

**Total = 1921**	**Participant characteristic**	**Prevalence of central obesity**
	**N (%)**	**% (SE)**
WC (cm)		
Not centrally obese^1^	652 (33.9)	-
Centrally obese^2^	1269 (66.1)	-
Age group		
60-69	875 (45.6)	63.2 (1.6)
70-79	749 (39.0)	69.4 (1.7)
80+	297 (15.5)	66.0 (2.8)
Area of residence		
Urban	996 (51.9)	72.7 (1.4)
Rural	925 (48.2)	58.9 (1.6)
Parity		
0	29 (1.5)	82.8 (7.1)
1-3	952 (49.6)	66.3(1.5)
4+	940 (48.9)	65.3 (1.6)
Current smoker		
No	1667 (86.8)	67.5 (1.1)
Yes	254 (13.2)	55.9 (3.1)
Currently consumes alcohol		
No	1849 (96.3)	66.3 (1.1)
Yes	72 (3.7)	59.7 (5.8)
Meat consumption		
< once/day	831 (43.3)	62.9 (1.7)
≥ once/day	1090 (57.1)	68.4 (1.4)
Fruit and vegetable consumption		
< once/day	87 (4.5)	58.6 (5.3)
≥ once/day	1834 (95.4)	66.4 (1.1)
Education level		
None	949 (49.4)	63.6 (1.6)
Any	972 (50.6)	68.4 (1.5)
Occupation group		
Agricultural	1048 (54.6)	61.3 (1.5)
Non-agricultural	873 (45.4)	71.8 (1.5)
Occupation by education level		
No education		
Agricultural	762 (80.3)	60.2 (1.8)
Non-agricultural	187 (19.7)	77.5 (3.1)
Any education		
Agricultural	286 (29.4)	70.3 (1.7)
Non-agricultural	686 (70.6)	63.2 (1.6)

^1^ Not centrally obese: WC < 80 cm.

^2^ Centrally obese: WC ≥ 80 cm.

### Separate effects of education and occupation on obesity

The top half of Table 3 shows the relationship between each SES indicator and central obesity. The unadjusted ORs indicated a positive association between each of education and occupation and central obesity (OR and 95%CI comparing those with any education *vs.* none: 1.24; 1.02, 1.50; and comparing those with a non-agricultural *vs.* agricultural occupation: 1.61; 1.33, 1.95). Adjustment of these estimates for age group and parity had little impact on the association. Further adjustments for health behaviours and area of residence resulted in the absence of evidence for an association between education and central obesity (1.19; 0.97, 1.45). However, adjustment for health behaviours did not have a large impact on the association between occupation and central obesity.

**Table 3:** Separate and joint effects of education and occupation on central obesity – Four Provinces Study, China (2008/09)

**Complete cases (N = 1921)**	**Unadjusted**	**Age group and parityadjusted**		**Age group, parity and health behaviour**^**2 **^**adjusted**			**Age group, parity, health behaviour and area of residence adjusted**	
	**OR (95%CI)**	***P*-value**^**3**^	**OR (95%CI)**	***P*-value**^**3**^	**OR (95%CI)**	***P*-value**^**3**^	**OR (95%CI)**	***P*cpvalue**^**3**^
**Separate effects**^**1**^								
Education level								
None	1		1		1		1	
Any	1.24 (1.02,1.50)	0.03	1.27 (1.05, 1.55)	0.02	1.19 (0.97, 1.45)	0.09	0.96 (0.78, 1.20)	0.7
Occupational status								
Agricultural	1		1		1		1	
Non-agricultural	1.61 (1.33, 1.95)	<0.001	1.59 (1.30, 1.94)	<0.001	1.46 (1.19, 1.81)	<0.001	1.11 (0.84, 1.45)	0.4
**Joint effects** (Odds of obesity for occupational status [Non-agricultural *vs.* agricultural] within education levels) ^**3**^								
Education level								
None	2.28 (1.57, 3.31)	<0.001	2.21 (1.52, 3.21)	<0.001	2.10 (1.43, 3.07)	<0.001	1.66 (1.11, 2.49)	0.01
Any	1.33 (0.99, 1.78)	0.06	1.25 (0.92, 1.70)	0.1	1.15 (0.84, 1.57)	0.4	0.84 (0.58, 1.20)	0.3
*P* for interaction^4^	0.02		0.02		0.02		<0.01	

^1^ Odds ratios of obesity for education level [Any *vs.* None] and occupational status [Non-agricultural *vs.* agricultural].

^2^ Health behaviours included current alcohol consumption, smoking status, meat consumption and fruit and vegetable consumption.

^3^*P*-value for the Wald test.

^4^*P*-value for the LR test comparing the models with and without the interaction term between education and occupation.

**Figure 3:**
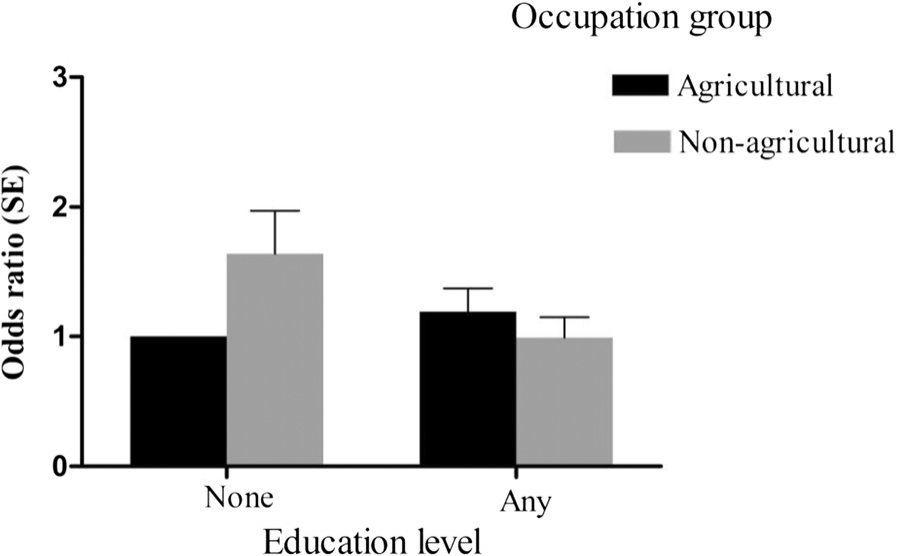
Graph of the interaction between education and occupation (adjusted ors* and 95%ci*).* Each bar represents the predicted odds in each group relative to the reference group (education = none; occupation = agricultural). The ORs are adjusted for age group, urban/rural residence, parity, dietary indicators, smoking and alcohol consumption.

Figure 3 shows the fully adjusted OR for central obesity for each combination of education level and occupation group. The pattern of estimates suggested that women with a non-agricultural occupation had higher odds of central obesity compared with women with an agricultural occupation if they had no education.

The bottom half of Table 3 displays the results for the regression models including the interaction term between education level and occupational status. There was statistical evidence for the interaction term between education and occupation (*P ≤* 0.02). The joint effect estimates show that having a non-agricultural *vs.* agricultural occupation was associated with higher odds of central obesity in women with no education (OR; 95%CI: 2.28; 1.57, 3.31 in the imputed data) corroborating the findings from Figure 3, but there was no evidence of such an association in the group of women with education. Adjustment for the additional covariates including health behaviours attenuated the magnitude of the OR estimate in women with no education.

### Sensitivity analysis

The analyses were repeated to explore the possibility that the interaction results were only significant due to measurement error resulting from differences in the overall educational levels of the occupational groups that were compared in the analysis (agricultural workers vs. non-agricultural workers including manual and office-based workers). Therefore, a sensitivity analysis was carried out after the exclusion of office-based workers from the non-agricultural occupation group so that the agricultural group was compared with the manual group – groups with more similar overall educational levels. The statistical evidence of an interaction was stronger (fully adjusted OR; 95%CI for obesity comparing manual workers with agricultural workers: 3.41; 2.03, 5.72 in the group with no education and 1.20; 0.84, 1,71 in the group with any education). This suggests that the results from the main analysis were conservative and valid.

## Discussion

This study investigated the hypothesis that education might protect against the obesogenic effects of having an occupation in the agricultural sector compared with having an occupation in the industrial and service sectors in a country undergoing rapid economic transition, by examining whether education modified the association between occupation and central obesity in a population of Chinese women. The results showed that having no education more than doubled the odds of central obesity associated with having a non-agricultural occupation compared with an agricultural occupation in this population (OR; 95%CI: 2.25; 1.55, 3.25). Differences in health behaviours including meat, fruit and vegetable consumption may have a role in explaining this. In women with any level of education no evidence of an association between occupation and obesity was found.

While the data are limited by their cross-sectional nature this study contributes new understanding to the SES-obesity association, demonstrating different associations with central obesity for two key SES indicators.

The need to examine the inter-relationships between SES more closely in transition settings. In high income countries, education, occupation and income levels tend to be collinear but this may not be the case in lower income countries. In particular, this study contributes to the growing evidence that education may have different properties as an SES indicator in relation to obesity in low- and middle-income countries compared with indicators that are more closely linked to material circumstances such as occupation and wealth [13,14].

### Comparison with prior studies

Many of the key SES-adiposity studies in lower income countries use multi-country data. Few have incorporated occupation as an SES indicator, favouring education and wealth instead as they are considered more reliable measures of SES in lower income contexts and, therefore, more comparable across time and space [34]. There are currently no studies in the epidemiological literature investigating the inter-relationship between education and occupation in relation to female obesity in lower income settings to our knowledge, although independent effects of education and wealth on obesity have been reported in single country studies. In Peru, the Philippines, China and Brazil [21,35-38] a positive association has been found between wealth and obesity together with an inverse or protective association between education and obesity (usually among women but not men) when both are taken into account in the analysis. Recent data from China corroborate the emergence of a protective association between education and obesity at least among urban residents [21] and women [22,23]. These patterns are comparable to findings in the early 1990s in Eastern Europe during its economic transition, when education and material circumstances acted differently as SES indicators of health outcomes [39].

Occupation has been considered to be a good measure of material circumstances [40] and found to be negatively associated with obesity in women (higher occupational status-lower obesity) [41] in high income countries. However, the nature of the association in low- and middle-income countries is likely to vary according to the specification of the occupational variable and the level of development of the country. The specification in this study was intended to capture the variation resulting from the differences between agricultural-based jobs and jobs in industry and services, in terms of the daily physical and food environment that affect health behaviours.

The results suggested that dietary behaviours including food and alcohol consumption accounted for part of the association between occupation and central obesity in the group with no education but it was not possible to fully assess the independent role of physical activity. Other studies from China suggest that diet may be more important than physical activity in explaining the association between occupation and markers of excess adiposity. In a study of 7,011 Chinese women 50 years and older examining the association between education and occupation (specified as manual vs. non-manual) and a composite index of the metabolic syndrome (including WC) adjustment for physical activity had little impact [42]. Another study [43] examining urban/rural differences in central obesity in 8,014 women attributed a higher proportion (43.8%) of the excess risk of central obesity in urban areas to diet and a lower proportion to physical activity.

The findings from the present study corroborate others in documenting the high levels of central obesity in China. Estimates from the China Health and Nutrition Survey from 1993 to 2009, representing a total of 52,621 participants, showed that there was a significant increase in central obesity in Chinese women 60 years and over – the age group examined in this study - from 47.4% (SE: 2.4) in 1993 to 66.5% (SE:1.3) in 2009 [20].

### Plausible and competing explanations

Excess adiposity is increasingly viewed as a mismatch between biology and the environment [44]. Economic transition to a higher income economy is usually associated with a move from a predominantly agrarian and/or subsistence economy to a predominantly industrial and/or service-based economy resulting in changes in dietary composition, occupational patterns and leisure time activities conducive to excess body fat storage [45-47]. But the mechanisms that explain the SES-adiposity association are complex and not fully understood [45]: the association may be bidirectional and confounded by other factors such as heredity, health behaviours and general socio-cultural norms [48], as well as show period variation [49-51].

At its most basic, higher status occupations might influence obesity risk through levels of physical activity [12], however they are also likely to be associated with living in an urban environment and, therefore, the consumption of higher levels of foods rich in fat and sugar and possibly lower leisure time physical activity. Furthermore, women’s entry into the labour force can lead to an increased reliance on processed or ready-made foods as well as a greater number of visits to restaurants and other prepared food outlets [9,52].

On the other hand, women’s education is known to be protective for a variety of health outcomes [15,16,53-55]. In the case of obesity, it may allow women to make better dietary and exercise choices through the cognitive advantages that can operate in a number of ways including improved access to and understanding of health related information, clearer risk perception related to lifestyle choices, altered time preferences and better self-control [17]. However, there is evidence that these cognitive advantages are unrelated to time-preferences and personality [16]. Alternative explanations include that more educated women may conform to different cultural norms of physical beauty that favour slimness [48,56] and that better education may operate through psychosocial pathways by affording better job-control and therefore lower stress levels which modulate inflammatory responses linked to obesity [57].

It is important to remember that countries undergoing rapid transition may experience an influx of new food products including high-calorie and nutrient-poor processed foods alongside other changes in lifestyle. In other words, the nature of disease risk changes. Yet public health infrastructure may not be equipped to deal with these nor engage the public in managing these risks. The combination of longstanding food insecurity, aggressive commercial marketing and inadequate public health systems may result in a large asymmetry of information between consumers and sellers when assessing consumption-related health risks. This could give those with higher education levels an advantage because they may be able to correct cognitive biases within this imbalanced information environment more easily.

### Implications of the findings

The main implication of the findings in this study is that obesity risk in low- and middle-income countries may not solely be determined by changing material circumstances associated with working and living in a different economic environment but that having a better level of education may protect against the detrimental effects of these significant changes in living conditions. This may occur through cognitive mechanisms that promote better dietary and leisure-time physical activity choices, and empower individuals in navigating new disease risks resulting from economic transition. Data from China show that the prevalence of obesity has increased at a faster rate in poorer rural areas than in richer urban areas [58] and that lower income groups have disproportionately increased their consumption of animal fat and edible oil and reduced their consumption of healthier traditional foods which may be a result of the penetration of global food corporations [59]. Improving education levels among these groups may contribute to improving health behaviours within the changing food environment experienced in rapidly changing economies like China. Evidence from Europe based on macro-level data show that national expenditure on education is inversely correlated with population levels of obesity [9]. Investments in education may be useful where legislation on commercial activity may be politically unfeasible, however, this should not be a substitute for the strengthening of public health systems and economic governance.

### Strengths and limitations

The Four Provinces data consisted of nationally representative data. It randomly recruited older women from four provinces in China, included anthropometric and health behaviour data, and had a high response rate. However, the cross-sectional nature of the data limits the interpretation of the findings in terms of temporal and causal inferences. The four provinces were broadly comparable and the overall sample had levels of central obesity comparable to national levels reported elsewhere. The prevalence of obesity was 66.0% (see Table 2) which was almost exactly the same as the prevalence reported for the age, sex and year equivalent group in the Chinese Health and Nutrition Survey which was 66.5% [20]. However, although the provinces had comparable levels of economic development and modernisation to other provinces, caution should be exercised in generalising our findings to all of China’s 169 million older inhabitants. The age range was confined to women over 65 years and limits the generalisation of the findings to the rest of the population.

There are many mechanisms that could explain the role of education which could not be explored due to the data limitations. For instance, the effect of body shape preference and early life deprivation could not be assessed, and inferences regarding the impact of health behaviours were limited due to the dichotomous specification of the variables. In terms of occupation, the non-agricultural category was heterogeneous and included both manual and office-based workers and could, therefore, be further segmented to examine dose–response or gradient effects as well as non-linear associations. However, this definition was informed by a substantial body of theory and empirical work documenting the link between shifts away from agriculture (towards an industrial economy) with a rise in obesity and attribute the rise to changes in diet and physical activity levels [7,60,61].

We excluded housewives from the sample as there was no theoretical basis underpinning the relationship between housewife status and obesity levels. This may have introduced bias in the analysis, however, the study did not aim to examine the association between occupational status and obesity but of the association between two specific occupational categories (agricultural and non-agricultural) in a context where there have been major shifts away from agricultural work and a parallel rise in obesity”.

Finally, the occupation variable may have been subject to reporting bias as those who classified themselves as having an agricultural occupation may have, in fact, been employed in other sectors as seasonal migrant workers. These issues require further exploration in the epidemiological literature examining socioeconomic status and non-communicable disease outcomes in lower income settings through improved data collection and measurement accuracy. Further investigation of the hypothesis and the mechanisms behind the observed associations would benefit from the use of longitudinal data.

## Conclusion

A common shortcoming of current SES-obesity studies is a restrictive uni-dimensional approach to socioeconomic status (SES), a shortcoming demonstrated with other health outcomes [55,62,63]. SES indicators (education, wealth, income, etc.) are interchangeably used without any explicit theorisation of their association with obesity or due consideration for the possible inter-relationships between these different dimensions of SES. This may leave epidemiology behind other disciplines that have sought to examine interactions between different SES indicators in relation to health [62,63]. The present study reports the existence of an interaction between two socioeconomic indicators on obesity in China and demonstrates a possible protective role of education in preventing the effects of economic transition resulting in changes in labour market opportunities for women. Further research could aim to elucidate potential mechanisms for this protective effect.

## Competing interests

The authors declare that they have no competing interest.

## Authors’ contributions

AA-S carried out the analysis and drafting of the manuscript. RC participated in the design and implementation of the data collection for the Four Provinces and in the drafting of the manuscript. MJS participated in the design of the statistical analysis and the drafting. MGM participated in the design and coordination of the study and helped to draft the manuscript. All authors read and approved the final manuscript.

## Pre-publication history

The pre-publication history for this paper can be accessed here:

http://www.biomedcentral.com/1471-2458/13/769/prepub
